# A NOVEL FRAMEWORK LINKING MNEMONIC AND HIPPOCAMPAL INTEGRATION TO LATE-LIFE REAPPRAISAL EFFICACY

**DOI:** 10.1093/geroni/igac059.2211

**Published:** 2022-12-20

**Authors:** Bruna Martins-Klein, Irina Orlovsky, Kristin Heideman

**Affiliations:** University of Southern California, Los Angeles, California, United States; University of Massachusetts Amherst, Amherst, Massachusetts, United States; Yale Child Study Center, New Haven, Connecticut, United States

## Abstract

Socioemotional theories suggest that surviving challenging experiences enhances emotional resilience with age, yet the role of memories is overlooked in most models of emotion regulation. In parallel, cognitive accounts focus on age-related memory deficits associated with overlapping hippocampal neural representations across unique memories (neural dedifferentiation). We propose a novel framework supporting enhanced late-life reappraisal via hippocampal dedifferentiation of memory representations across current and past experiences. We review classic studies supporting mood benefits from integrated positive narratives following adverse autobiographical events. We also discuss multivariate neuroimaging evidence supporting overlapping hippocampal representations and ventromedial prefrontal cortex (vmPFC) involvement in meaning-making processes. We posit that greater hippocampal dedifferentiation across life memories may facilitate associative binding of current and past stressors as well as reappraisals in vmPFC. This process may provide avenues for generalizing past reappraisals to novel contexts and reducing cognitive demands of reappraisal. In addition, we discuss the possible age-related facilitation of this process, as a greater number of life experiences may become increasingly integrated with one another over the lifespan. These integrated neural associations may serve to make reappraisals from the past more readily accessible and applicable in new contexts over time, increasing routes to positive narratives following stress. We discuss future directions for testing components of the proposed model using multivariate neuroimaging methods. We conclude by briefly reviewing the possible clinical impact of mnemonic emotion regulation in promoting emotional well-being among older adults, using a strengths-based approach that leverages wisdom from experience and neural processes facilitated with age.

